# Regarding: ‘High-intensity-focused ultrasound in the treatment of primary prostate cancer: the first UK series’

**DOI:** 10.1038/sj.bjc.6605453

**Published:** 2009-12-08

**Authors:** T S Clark

**Affiliations:** 1 Carlisle, UK

**Sir,**

Two of the statements within the abstract of this paper (Ahmed *et al*, 2009) have been seized upon (and misinterpreted) in a recent press release and subsequent press articles. The misinterpretations have arisen from an unfortunate lack of precision in the wording of the abstract. As there has been so much press coverage, and as patient recruitment to further HIFU studies is occurring now on the site bearing the press release, I feel it is important that the ambiguities of the abstract are discussed and corrected.

The two abstract claims (with ambiguous wording in bold) are these:

1. **‘Overall** there was no evidence of disease after **one HIFU session** in 92.4% (159 of 172) of patients’ – the time after operation of this measurement is not stated. Press release and press articles uniformly assume that it was at 12 months.
2. ‘Potency was **maintained in 70%** by 12 months’ – the patient population on which this was measured is not stated in the abstract. Press release and press articles assume that it was the whole group (and wrongly deduce that only 30–40% of the measured group were impotent at 12 months).

First, ‘92% no evidence of disease etc’: The statement appears as a summary of the results. The data for this measurement point are not explicitly presented in the abstract or paper. This makes it impossible to be certain of the time of measurement.

From the size of the patient group (172 patients, i.e., all patients) this would appear to be a very early measurement point (shortly after the operation). This can be inferred from the data in Table 3 (p.22) and the Kaplan–Meier curves (p 23). These show data for fewer patients at each successive milestone (lost to follow-up, died or not yet reached the milestone), with data for only 155 patients at 3 months. It is not evident which test is used for ‘evidence of disease’ at this early measuring point – possibly PSA⩽0.5 ng ml^−1^ (units of *μ*g ml^−1^ appear in the abstract but nowhere else). It is particularly unfortunate that early data have been highlighted without it being noted that it is early data. In addition, the use of such early data as an ‘overall’ summary of performance does not seem to be appropriate, particularly as the later data (78% of 83 patients show no evidence of disease at 12 months) are also given in the abstract, and thus seem to be contradicted by the summary.

Second, ‘70% maintain potency etc’: It is slightly difficult to follow from the paper how many were in the group assessed, possibly due to a typo. However, the statement appears to be based on results from 34 patients at 12 months, of whom 16 were potent at 12 months whilst 24 out of 51 patients were potent before the procedure. Thus, 16 out of 24 gives 66.7% maintaining potency (p 21). The text of the paper mentions that potency questionnaires were available for 49 patients at 12 months (p 20); only 34 (or is it 51?) appear in the data set analysed at 12 months (p 21).

The rates of return of questionnaire between the groups ‘potent before procedure’ and ‘impotent before procedure’ are not addressed but could potentially introduce some bias. Even if data are available for every patient who has reached the 12-month milestone, the lack of information on Viagra use (before and after) would make it impossible to assess how much impotency has been caused by the procedure. In all, it appears very unsafe to extrapolate the potency results from this small sample to the whole test population. The authors do not do so; the error of the press in doing so might have been avoided if the authors had specified the sample size in the abstract and presented more comment on the reliability of these specific data.

Both these claims and their subsequent misrepresentations in the press have been used to make invalid comparisons with other more conventional treatments. Patients volunteering for HIFU should do so in the light of full and correct information. I would urge the authors to make public the necessary clarifications.
